# Endocrine Regulation of Lifespan in Insect Diapause

**DOI:** 10.3389/fphys.2022.825057

**Published:** 2022-02-15

**Authors:** Corinne Hutfilz

**Affiliations:** Department of Molecular Biology, Cell Biology and Biochemistry, Brown University, Providence, RI, United States

**Keywords:** diapause, endocrine, hormone, lifespan, aging, longevity, ecdysone, insulin

## Abstract

Diapause is a physiological adaptation to conditions that are unfavorable for growth or reproduction. During diapause, animals become long-lived, stress-resistant, developmentally static, and non-reproductive, in the case of diapausing adults. Diapause has been observed at all developmental stages in both vertebrates and invertebrates. In adults, diapause traits weaken into adaptations such as hibernation, estivation, dormancy, or torpor, which represent evolutionarily diverse versions of the traditional diapause traits. These traits are regulated through modifications of the endocrine program guiding development. In insects, this typically includes changes in molting hormones, as well as metabolic signals that limit growth while skewing the organism’s energetic demands toward conservation. While much work has been done to characterize these modifications, the interactions between hormones and their downstream consequences are incompletely understood. The current state of diapause endocrinology is reviewed here to highlight the relevance of diapause beyond its use as a model to study seasonality and development. Specifically, insect diapause is an emerging model to study mechanisms that determine lifespan. The induction of diapause represents a dramatic change in the normal progression of age. Hormones such as juvenile hormone, 20-hydroxyecdysone, and prothoracicotropic hormone are well-known to modulate this plasticity. The induction of diapause—and by extension, the cessation of normal aging—is coordinated by interactions between these pathways. However, research directly connecting diapause endocrinology to the biology of aging is lacking. This review explores connections between diapause and aging through the perspective of endocrine signaling. The current state of research in both fields suggests appreciable overlap that will greatly contribute to our understanding of diapause and lifespan determination.

## Introduction

Diapause is a physiological adaptation to adverse conditions meant to increase the odds of an organism’s survival into reproductive age (Karp, 2021). Traits of diapause include extended lifespan, slowed development, altered metabolism and body composition, delayed reproduction, and increased hardiness against environmental threats. Some insects exhibit a programmed period of diapause during development (obligatory diapause), while others use environmental cues such as nutrient availability, temperature, humidity and photoperiod, to determine whether it is appropriate to delay development until favorable conditions appear, or whether the current environment will support the energy-intensive process of normal development. The latter mode is named “facultative diapause.” In cases where diapause phenotypes are milder, fewer, easily reversed, or induced quickly as part of a generalized stress response, other terms such as “quiescence,” “dormancy” or “torpor” are sometimes used (Zonato et al., 2017; Karp, 2021). While functionally distinct, variations in diapause or diapause-like physiology are worthwhile to study in the context of longevity for their evolutionary and mechanistic similarities.

**FIGURE 1:**
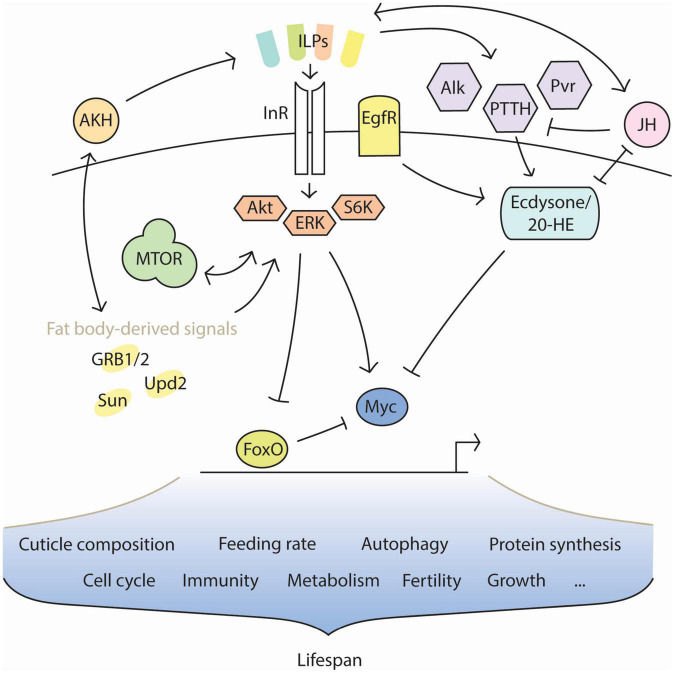
Interaction of key factors in diapause. Insulin signaling profiles change in diapause, caused by the activity of different insulin-like peptides (ILPs). The ILP profile must remain dynamic in diapause to support the diapause-associated metabolism over time and changing conditions. Other factors, such as the nutrient-sensitive adipokinetic hormone (AKH) and the pro-reproductive juvenile hormone (JH), influence insulin signaling and are themselves influenced in turn. JH opposes the metamorphic action of ecdysone and its upstream regulators. FoxO is typically inhibited by insulin signaling, but is disinhibited during diapause. Conversely, the pro-growth transcription factor Myc is typically promoted by insulin signaling, but becomes inhibited during diapause. Many features of diapause physiology are regulated through interactions between insulin signaling, JH, ecdysone, and nutritive feedback signals from the fat body. The extended lifespan of insects in diapause is likely a product of these features’ combination.

The transmission of information about the environment occurs through a complex network of competing endocrine signals. Though the hormones involved are largely homologous across species, the precise endocrine program of diapause has remained elusive due to its branching nature; no individual hormone is regulated apart from any other, and there is extensive overlap in the downstream molecular targets of each (Figure 1). It is therefore difficult to attribute any given effect of diapause to one hormone by itself. However, it is equally difficult to consider the complete breadth of diapause endocrinology in any one study. This review seeks to assist by drawing connections between results from recent literature. Adding to the intricacy of this task is the fact that diapause consists of several phases. Preparation, initiation, maintenance, and termination are mediated by separate endocrine states. Recent advances in diapause research show that each phase consists of transcriptionally distinct sub-phases (Kubrak et al., 2014; Li et al., 2015), as well. Much work in the field of insect diapause has focused on mechanisms of diapause initiation. Specifically, much attention has been granted to mechanisms responsible for the induction of early diapauses between larval or pupal transitions. Reproductive dormancy, characterized by developmental regression of the gonads, has also been well-studied as a proxy for the complete diapause phenotype in adults.

Studies in diapause often involve perturbation of known elements of diapause physiology—pathways such as insulin/insulin growth factor signaling (IIS) or ecdysone biosynthesis—or modification of tissues responsible for these hormones’ secretion. Laboratory disruptions in the precisely timed endocrine program coordinating normal development can result in partial or complete induction of diapause. The degree to which partial diapause phenotypes are induced without exposure to diapause-inducing environmental stimuli, or the degree to which these phenotypes are ameliorated upon environmental exposure, is usually taken to indicate something about the importance of the hormone in question. Nearly all cases extend lifespan. Lifespan extension is robustly conserved among insect diapauses across developmental phases, as it enables the organism to persist through unfavorable seasons that typically last much longer than the organism’s normal lifespan. This trait is slowly emerging as a promising model system to study mechanisms of lifespan plasticity outside the immediate context of insect diapause. Diapause represents a fascinating adaptation wherein the median lifespan of a species dramatically changes in a reversible manner. The conservation of this trait across taxa, experimental mode of diapause induction, and developmental stage suggests that lifespan is not a static trait programmed per species, but is determined by integrating external information with processes that are fundamental to most organisms. Understanding what controls the diapause-associated change in lifespan will help answer the question of why we age at the rate that we do.

Many findings in the aging biology field underpin longevity with changes in pathways that mimic the way those pathways are naturally modified during diapause. For example, it has long been observed that manipulations of IIS extend lifespan across species (Sim and Denlinger, 2013). IIS-dependent lifespan extension is usually accompanied by other diapause-like phenotypes, such as resistance to infection and loss in reproductive capacity. Although increasing evidence finds ways to uncouple these phenotypes, it is unlikely that they are co-induced by coincidence. Rather, it may be the case that activation of one facet of diapause signals a systemic “diapause response” to perceived stress, which in turn activates supporting pathways. Other modes of lifespan extension (manipulations in neuronal signaling, amino acid metabolism, dietary restriction, and more) also invoke diapause physiology (Suo et al., 2009; Katewa et al., 2012; Itskov and Ribeiro, 2013; Kapahi et al., 2017; Ling and Raikhel, 2018; Parkhitko et al., 2020). These results from aging biology can be simultaneously united and explained by considering them in the context of diapause. Doing so will benefit both fields by expanding the relevance of conclusions from either. Therefore, this review is written from the perspective that diapause represents the evolutionary context for laboratory manipulations of lifespan.

### Endocrine in Diapause

As a mechanism that evolved to modify development, the onset of diapause demands changes to the endocrine program that defines normal progression through molts to reach reproductive adulthood. Historically, the regulation of endocrine has been characterized in the context of development. Less is known about how the entrance or maintenance of diapause imposes upon developmental hormones, but especially in the case of adult diapause, this topic is beneficial for understanding the role of early development in lifespan plasticity. Examination of the work done in development is informative for speculating about how endocrine functions and changes course in diapause and longevity.

## Ecdysone

The steroid hormone ecdysone is well-known to catalyze metamorphosis between developmental stages and antagonize growth. In holometabolous insects, growth and metamorphosis are distinct processes. Growth is defined by the accumulation of mass, cell division, and increase in body size, whereas metamorphosis refers specifically to the differentiation of prime tissues into developmentally terminal ones. The large upregulation of ecdysone at the end of each molt signals the organism’s pausing of growth and the initiation of metamorphosis into the next stage. This period is called “critical weight,” which means the molt has accumulated enough mass to signal entrance into the next stage of development. In pre-adult molts such as larvae or nymphs, ecdysone is synthesized and secreted from the prothoracic gland (PG). The PG is part of the larger complex of endocrine signaling glands adjacent to the brain called the ring gland. Ecdysone is released into the hemolymph, which distributes the hormone to peripheral tissues where it is imported via the ecdysone importer (EcI) (Okamoto et al., 2018). Once imported, ecdysone can differentially regulate transcription depending on whether it is converted to 20-hydroxyecdysone (20HE); both forms have been shown to have active effects, though fewer studies have investigated the biological relevance of ecdysone signaling before its conversion (Beckstead et al., 2007; Tanaka, 2013; Yamanaka et al., 2013; Ono, 2014). Conversion to 20HE occurs through the activity of Shade, one enzyme derived from the ‘Halloween’ group of genes responsible for the complete ecdysone biosynthetic pathway (Petryk et al., 2003). Briefly, precursor ecdysone is derived from nutritionally acquired sterols and is modified through a series of early oxidation steps terminating in a chain of cytochrome P450 monooxygenases. For a thorough examination of the ecdysteroid biosynthetic process, see Niwa and Niwa (2014).

The binding of 20HE to the nuclear ecdysone receptor (EcR) induces the receptor’s entrance into the nucleus and subsequent heterodimerization to Ultraspiracle (Usp) (Yao et al., 1992, 1993). The complex then binds DNA to mediate the transcription of ecdysone response genes that catalyze metamorphosis, such as the Broad-Complex, E93, and EcR itself (Karim et al., 1993; Lee et al., 2000; Ghbeish et al., 2001). It has been shown that the set of genes regulated by nuclear EcR differs according to developmental stage, though it remains unclear how the organism measures developmental progress to modify the transcriptional targets of the same receptor complex across stages (Yamanaka et al., 2013; Uyehara and McKay, 2019). Apart from binding to ecdysone response elements, EcR also affects transcription through changes in chromatin structure (Ashburner, 1973). Chromosome puffing—sites of decompressed chromatin that facilitate transcription—has been observed in a variety of insects in the presence of ecdysone (Stocker and Pavan, 1977; Ashburner, 1990; Russell and Ashburner, 1996; Gariou-Papalexiou et al., 2001). As the genes regulated by EcR depend on developmental timing, so do the genomic loci of puffs. This decompaction is traceable at least in part to the EcR cofactor SMRTER, whose activation leads to repression of the histone deacetylase Rpd3 (Tsai et al., 1999). Other transcription factors, such as the EcR-Usp complex itself, may induce similar structural changes through the recruitment of RNA polymerase-associated chromatin binding proteins.

Our understanding of the effectors of ecdysone signaling are still incomplete, however. Recent work has suggested involvement of Thor, a 4E-binding translation initiation factor. Thor is an effector of insulin/insulin-like signaling (IIS) and the mechanistic Target of Rapamycin (mTOR) pathway that each regulate the intersection of lifespan and nutrition (Kapahi et al., 2004; Herboso et al., 2015). Wing disks from third instar, ecdysone-deficient larvae have increased expression of *Thor*, suggesting that ecdysone plays a repressive role against transcription of the *Thor* gene product. Indeed, while insulin-like peptide (ILP) secretion is unaffected in these larvae, many tissues show impaired growth. This result complicates the long-held idea that the growth-inhibitory effects of ecdysone are inversely correlated with pro-growth IIS and mTOR signaling (Rusten et al., 2004; Colombani et al., 2005).

The picture becomes cloudier yet from the perspective of age and adulthood. As the PG no longer exists in adult insects, ecdysone synthesis originates from other tissues. In *Drosophila*, evidence suggests adult ecdysone synthesis takes place in the gut, muscle, Malpighian tubules, and male accessory glands, at least (Hentze et al., 2013; Zheng et al., 2018). This variety of locations may indicate an adult capacity to respond to environmental stress in a tissue-specific manner. Increasing 20HE titers no longer initiates metamorphosis in adults; instead, 20HE responds to stresses such as nutritional deficiency, water loss, or heat (Nghiem et al., 2000; Rauschenbach et al., 2000; Gibbs and Markow, 2001; Terashima et al., 2005; Zheng et al., 2018). These effects are both more common and more damaging in old age, and also represent stressors that the physiological adaptations of diapause have evolved to protect against. Accordingly, it has been shown that ecdysone increases with age and induces expression of peptidoglycan recognition protein-LC (PRP-LC), a critical component of immune signaling needed to respond against infections that are expected under suboptimal environmental conditions (Rus et al., 2013; Zheng et al., 2018). Increasing ecdysone in older adults also causes fibrosis in a manner dependent on epidermal growth factor receptor (EgfR) and dopamine-EcR, a receptor that binds ecdysone before its conversion to 20HE. Results from this work suggest ecdysone continues its metamorphic role in adults, but has lost the tight regulation required for development (Zheng et al., 2020). It follows that heterozygous mutation for EcR extends adult lifespan (Simon et al., 2003). Presumably, these flies evade some of the fibrosis expected with age and do not aberrantly induce age-associated inflammation through PRP-LC (Garschall and Flatt, 2018). This occurs in a manner independent of reproductive capacity.

Extrapolating from its role in development, perhaps ecdysone responds to stress in adults by inducing more subtle changes to the organism that benefit survival and longevity. One such modification may be alteration of the cuticle composition. The cuticle serves several functions to protect against stress: just after eclosion, cuticle hardening helps resist mechanical forces such as punctures or tears, which could lead to infection (Noh et al., 2016). The cuticle also undergoes a tyrosine-dependent tanning process through the deposition of various pigments such as melanins or tetrapyrroles (Shamim et al., 2014; Solano, 2014; Noh et al., 2016). Pigmentation is an important step in the final stage of development to resist damage from UV radiation, to which the insect is especially vulnerable during winter or drought when shade is sparse from failing vegetation. Increased sunlight exposure also causes water loss, leading to dehydration and inflammation, which are exacerbated with age (Franceschi et al., 2018; Zheng et al., 2018). The function of water retention is controlled by lipid barriers deposited throughout the cuticle (Lockey, 1988). As the organism ages, these barriers deteriorate and change composition (Gibbs et al., 1991; Nghiem et al., 2000; Kuo et al., 2012). Therefore, the age-associated increase in 20HE titers may act as a compensatory mechanism intended to remodel the cuticle to defend against the damaging effects of water loss caused by deterioration of cuticle lipid barriers.

The expression patterns of ecdysone throughout the different stages of diapause are largely unknown across species. However, there is evidence from flesh flies and moths to suggest entrance into diapause involves an initial increase in ecdysone, perhaps as a stress response similar to that seen in aging, where ecdysone is elevated in anticipation of the drought conditions associated with diapause season(s) (Denlinger et al., 1997; Rinehart et al., 2001). An initial burst of ecdysone may serve to drive the generation of alternative, diapause-specific structures in young, diapause-destined adults, such as the further tanning of the cuticle and restructuring of its lipid composition that have already been observed (Denlinger, 2002; Li and Denlinger, 2009). Other insect species, however, initiate diapause with a drop in ecdysone titers (Denlinger et al., 2012; Guo et al., 2021). While these observations do not preclude the suggestion that ecdysone induces diapause-specific structures in later developmental stages, it is worth noting that the functions of ecdysone—and endocrine more broadly—are poorly conserved.

In conclusion, it may be the case that ecdysone has significant effects on lifespan depending on its time of expression: during metamorphosis and diapause, elevated titers of ecdysone affect the insulin signaling profile to downregulate pro-aging factors like Myc and activate anti-aging factors like the Forkhead box-class O transcription factor (FoxO). Additionally, ecdysone could function in diapause to catalyze the metamorphosis of structures that elongate lifespan through resistance against stresses that exacerbate aging. However, there is currently no direct evidence as to whether these structures in diapause contribute to longevity, or whether their generation depends on ecdysone. It is possible that their effects are limited to survival; without them, the organism might be incapable of withstanding the harsh conditions expected during diapause.

## Insulin

Diapause-associated modifications to IIS cause physiological changes that often mirror those reported in studies examining the relation between IIS and aging (Garsin et al., 2003; Kramer et al., 2003; Broughton et al., 2005; Sim and Denlinger, 2008; Kubrak et al., 2014; Altintas et al., 2016; Cambron et al., 2021; Karp, 2021). Indeed, these changes are also derived from similar molecular events, such as the tissue-specific activation of the growth-inhibitory factor FoxO, upregulation of Toll-dependent innate immunity, and increased expression of adipokinetic hormone (AKH), the functional homolog to mammalian glucagon (Kubrak et al., 2014; Suzawa et al., 2019). Correspondingly, insofar as ecdysone signaling mediates IIS as well, many of these characteristics are also observed in the context of metamorphosis (Davidson and Swalla, 2002; Hughson et al., 2021). In both metamorphosis and diapause, modification of IIS correlates with a reduction or cessation of feeding, concurrent with the antagonism of growth and aging. These similarities warrant exploration of the idea that laboratory manipulations of IIS do not extend lifespan without evolutionary precedent, but recapitulate elements of the metabolic and reproductive programs of diapause and metamorphosis, which present as slow aging, low fecundity, and stress resistance.

### Insulin and Ecdysone

The deceleration of growth coupled to metamorphosis is achieved through ecdysone-dependent inhibition of the mitogenic transcription factor Myc (Delanoue et al., 2010; Rajan and Perrimon, 2010). Myc is repressed by FoxO, which is in turn inhibited by IIS. Little is known about the exact mechanism by which ecdysone inhibits IIS to activate FoxO. In *Helicoverpa armigera*, recent work provides evidence that 20HE acts upstream of IIS to maintain InR in a dephosphorylated state (Li et al., 2021). Because ecdysone has strong effects in the fat body, it is also possible that ecdysone downregulates IIS during metamorphosis by inhibiting one or more of the fat body-derived growth signals. These signals will be described later in this review for their interaction with IIS under variable nutrient conditions that contribute to diapause (Colombani et al., 2005). In sum, more research is needed to understand this pathway in its entirety, but it is clear that ecdysone is one among many regulators of IIS (Ahmad et al., 2020).

In turn, IIS controls the production of ecdysone. Increasing or decreasing IIS during development alters the time to critical weight and the consequent timing of expression of ecdysone synthesis genes (Rulifson et al., 2002; Shingleton et al., 2005; Walkiewicz and Stern, 2009). Perturbations in nutritional sensing via mTOR or IIS limits the growth of the PG, causing increased larval body size (an extension of the growth period), and delayed ecdysis (Gu et al., 2009; Kemirembe et al., 2012). Downstream of IIS, extracellular signal-regulated kinase (ERK) can also induce activity of the transcription factor Pointed, which modifies the EGF receptor signaling pathway (O’Neill et al., 1994; Traverse et al., 1994; Morimoto et al., 1996). Among other functions, EGFR signaling regulates the production of ecdysone in the PG (Cruz et al., 2020; Pan and O’Connor, 2021).

### Insulin and Nutrition

During growth phases, insulin peptide synthesis and secretion are regulated in response to nutritional cues acquired through dietary amino acids, sugars and carbohydrates (Kim and Neufeld, 2015). Insulin producing cells (IPCs) of insects are most prominently studied in the brain and fat body, but IPCs exist in many other tissues throughout the organism, such as the digestive and reproductive systems (Nässel et al., 2013). IIS regulates lifespan and tissue growth by sensing the availability of metabolites both directly and indirectly (Colombani et al., 2003; Fridell et al., 2009; Kréneisz et al., 2010; Rajan and Perrimon, 2012; Park et al., 2014). In the brain, exogenous glucose is sufficient to stimulate fluctuation in intracellular IPC Ca^2+^, causing secretion of insulin peptides (Kréneisz et al., 2010). Similarly, amino acids can pass through the gut lumen to signal directly to tissues via hemolymph circulation (Holtof et al., 2019). Indirectly, the import of leucine via the IPC-bound transmembrane proteins Juvenile Hormone-Inducible-21 (JhI-21) or Minidiscs (Mnd) is also sufficient to cause insulin secretion (Manière et al., 2016; Ziegler et al., 2018). In the gut and fat body, amino acids are imported via Mnd and Slimfast (Slif) to initiate multiple modes of insulin regulation that are largely dependent on secreted factors. The amino acid sensing folliculin/folliculin-interacting protein complex activates mTOR in the fat body through a series of RAG GTPases (Tsun et al., 2013; Shimobayashi and Hall, 2016). Once translocated to the lysosome, active mTOR modifies IIS by acting on downstream components like S6 kinase (S6K), Akt and FoxO (Zhang et al., 2011; Sangüesa et al., 2019). Evidence also exists for mTOR regulation of IPCs upstream of the insulin signaling cascade, though these studies are mainly focused in mammalian models (Gu et al., 2011; Alejandro et al., 2017; Sangüesa et al., 2019). Another mode of indirect insulin regulation in the fat body comes from the ligand Stunted (Sun). The receptor for Sun, Methuselah (Mth) (Delanoue et al., 2016), is expressed in the IPCs. Amino acids stimulate the release of Sun from the fat body and allow it to circulate to the brain via hemolymph, where Sun binds Mth to induce the release of IPC-derived ILPs; it follows that mutation of Mth extends lifespan via downregulation of IIS (Lin et al., 1998). Fat body-derived growth blocking peptides (GRB1, GRB2) are also capable of regulating IIS. Secreted GRB1 binds an epidermal growth factor (EGF) receptor on inhibitory neurons innervating the IPCs (Koyama and Mirth, 2016; Meschi et al., 2019). Although originally defined as growth inhibitors, GBPs encourage growth by repressing the activity of inhibitory neurons, thereby promoting the secretion of ILPs from the innervated IPCs (Hayakawa, 1991). Furthermore, the presence of sugars or carbohydrates in the fat body stimulates the release of CCHamide-2 (CCHa2) and Unpaired 2 (Upd2) that each modify IIS in the brain (Rajan and Perrimon, 2012; Li et al., 2013; Ren et al., 2015). There are numerous non-redundant mechanisms by which insulin is regulated before its secretion, and there are likely even more that extend beyond what we currently understand (Teleman, 2009; Post and Tatar, 2016; Ahmad et al., 2020; Manière et al., 2020; Chowański et al., 2021; Ling and Raikhel, 2021). Non-nutritional cues and endogenous insulin decoys can modify IIS after secretion to generate more specialized physiological and behavioral responses (Sloth Andersen et al., 2000; Siddle, 2012; Stefanatos et al., 2012; Okamoto et al., 2013; Barber et al., 2016). In species with multiple ILPs, each peptide signals in a distinct manner whose targets are not yet fully known (Post et al., 2018; Yamamoto et al., 2021). Downstream effects of IIS are additionally modified by tissue-specific levels of intracellular IIS targets, as well as competing signals from other pathways that intersect with IIS effectors (Siddle, 2012; Ruzzi et al., 2020). For these reasons, IIS is best construed as a highly flexible pathway. The phenotypes observed via disruptions in individual components likely represent real world scenarios in which the pathway is modified to produce a programmed response to some environmental cue.

After secretion into the hemolymph, ILPs bind the insulin receptor (InR) in peripheral tissues and cause the receptor to autophosphorylate (Zhang et al., 2011; Sim and Denlinger, 2013). Recruitment and phosphorylation of the insulin receptor substrates Lnk and Chico then initiates a phosphorylation cascade dependent on the kinases phosphatidylinositide 3-kinase (PI3K), Akt, and ERK (Yenush et al., 1996; Werz et al., 2009; Zhang et al., 2011; Almudi et al., 2013). Activation of Akt leads to the inhibition of FoxO, which then promotes cell proliferation through the disinhibition of Myc and the inhibition of Thor and several negative regulators of mTOR signaling (Koyama et al., 2013). Akt also promotes energy storage through the activation of glycogen synthase kinase-3 (GSK-3), which opposes the tissue-autonomous, energy mobilizing action of glycogen phosphorylase (GlyP) (Yamada et al., 2018). The downstream effects of ERK signaling are diverse and highly dependent on crosstalk with other pathways, encapsulating functions such as cell division, differentiation and apoptosis (Traverse et al., 1994; Shaul and Seger, 2007; Zhang et al., 2011).

### Insulin Peptides

Most insects have multiple ILPs with varying degrees of structural and functional redundancy. Accordingly, ILPs are regulated in manners both concerted and individual, depending on stimulus. Much work has been done to establish the diapause-like phenotypes of IPC-ablated flies (Rulifson et al., 2002; Broughton et al., 2005; Haselton et al., 2010; Ojima et al., 2018). The functions of ILPs are best studied in *Drosophila* and will be summarized here for their roles in aging and diapause, keeping in mind that while individual ILPs may not be conserved across taxa, the regulatory framework is broadly applicable: environmental stimuli induce a subset of ILPs with specialized functions appropriate for the given conditions.

#### Insulin-Like Peptide 2

ILP2 is the most abundant and extensively characterized of the *Drosophila* ILPs and is most similar to mammalian insulin in that its sequence is most homologous and its activity is primarily (but not exclusively) glucose-dependent (Brogiolo et al., 2001; Buch et al., 2008; Géminard et al., 2009; Kim and Neufeld, 2015; Post and Tatar, 2016). ILP2 chiefly regulates the production of glycogen by maintaining the phosphorylation state of glycogen phosphorylase (GlyP) (Post et al., 2018). Buch et al. (2008) revealed that the lifespan extension of IPC-ablated flies is partially rescued through expression of *ilp2* alone, supporting a central role of ILP2 in mediating IIS-dependent longevity. However, conflicting results challenge the assertion that ILP2 is sufficient to control lifespan. RNAi knockdown of *ilp2* resulted in flies with lifespan similar to that of wildtype flies of the same genetic background, whereas in a separate study from the same group, null mutation of *ilp2* resulted in modest extension with statistical significance (*p* < 0.001) (Broughton et al., 2008; Grönke et al., 2010). These conflicting results are possibly attributable to the many interacting pathways of ILP signaling that affect physiology in ways we do not yet understand. Changes in the level of a single ILP causes change in the levels of other ILPs, as well as changes in how the organism responds to temperature, diet, and even commensal bacteria (Broughton et al., 2005; Grönke et al., 2010; Bai et al., 2012; Chowański et al., 2021; Ling and Raikhel, 2021; Semaniuk et al., 2021). Correspondingly, though mRNA levels of *ilp2* and *ilp5* correlate with the inducibility of reproductive diapause, these findings were temperature-dependent, suggesting ILP levels alone do not control diapause physiology, but are likely an important facet of the larger diapause program (Schiesari et al., 2016).

#### Insulin-Like Peptide 6

The IGF-like ILP6 was first characterized in the fat body, but sites of production also include the gut, salivary glands and surface glia (Brogiolo et al., 2001; Okamoto et al., 2009; Chell and Brand, 2010; Okamoto and Nishimura, 2015). ILP6 is known to promote growth during periods of starvation, including both the natural non-feeding larval-pupal transition, as well as imposed starvation conditions outside this stage, such as diapause (Slaidina et al., 2009; Kubrak et al., 2014). Interestingly, expression of *ilp6* alone is sufficient to induce phenotypes associated with diapause, such as decreased fecundity, increased concentration of energy storage molecules and extended lifespan. *ilp6* is also positively correlated with *ilp5*, an ILP that responds to amino acids via mTOR in surface glia (Okamoto and Nishimura, 2015). Both are required for sustained growth under limited nutrient availability. The lifespan extension of *ilp6* overexpression mutants may be attributable to the mutants’ downregulation of *ilp2*; loss of *ilp2* has been shown to increase lifespan in a *ilp1*-dependent manner (Grönke et al., 2010; Post et al., 2019). However, *ilp1* overexpression in an otherwise wildtype background does not extend lifespan, leaving the question open as to how ILPs like ILP1 and ILP6 function in a paradoxically anti-aging mechanism.

#### Insulin-Like Peptide 1

*ilp1* is typically expressed during the pupal stage and remains repressed for the duration of adulthood (Slaidina et al., 2009). During the onset of diapause, however, *ilp1* becomes highly expressed and remains so for several weeks (Liu et al., 2016). Little is known about the downstream mechanism of ILP1. Still, the convergence of its roles in diapause and lifespan extension support the idea that diapause represents the natural context for longevity achieved through manipulations of IIS. Indeed, in a study examining the relationship between taste perception and longevity, Ostojic et al. (2014) found that taste-impaired female flies exhibit longer lifespan and elevated transcripts for *ilp1, ilp3*, and *ilp6* in a *FoxO*-dependent manner. Taste is a sensory modality similar to olfaction used to determine the availability of nutrition. With this understanding, these results could imply that ILP1 responds to starvation, as one environmental cue signaling the onset of winter, to induce the metabolic program of diapause that supports longevity.

The metabolism of diapause typically relies on an abundance of lipid storage and circulating trehalose, but less is known about the molecular changes that bring about this state. As a diapause-specific ILP, ILP1 is an attractive candidate to regulate the induction of the diapause metabolism upstream, and will be expanded upon here. In the absence of *ilp2, ilp1* induces AKH (Post et al., 2019). AKH has been reported to extend lifespan (Waterson et al., 2014). However, *ilp1* overexpression does not extend lifespan. This discrepancy suggests a missing mediator between AKH and its effects on longevity. The usual context of AKH signaling is during periods of starvation or water loss. Perhaps the pro-longevity effect of *ilp1* does not result from its expression alone, nor its regulation of AKH, but may be mediated through conditions closer to those that signal diapause. Under this model, the pro-longevity metabolism of diapause may depend on unknown factors that mediate between AKH and the perception of nutrition. One molecule that could play this role is trehalose, as a nutritive sugar that is upregulated in response to the cold conditions expected in *Drosophila* diapause. Indeed, though AKH is traditionally considered a starvation-responsive hormone, Kim and Neufeld (2015) demonstrate that AKH responds to sugars, as well (Doğan et al., 2021). This suggestion is further supported by the fact that AKH stimulates trehalose production, generating a putative feedback loop in diapause to support both longevity and cryo-protection (Isabel et al., 2005). Other candidate mediators include fat body-derived signals, whose effects are incompletely understood. Particularly, Upd2 is known to both regulate AKH secretion and respond to high sugar levels (Post and Tatar, 2016; Zhao and Karpac, 2017). Upd2 is also a known regulator of ILP secretion in the IPCs, where ILP1 is produced (Rajan and Perrimon, 2012). However, in a time course study examining *Drosophila* diapause, Upd2 expression was found to trail after *ilp1* expression (Kubrak et al., 2014). Upd2 is also known to oppose FoxO and promote of the release of ILP2, discrediting the idea that Upd2 supports diapause longevity during the induction phase (Rajan and Perrimon, 2012; Zhao and Karpac, 2017; Lourido et al., 2021). It is possible that another starvation associated, fat body-derived signal supplies the missing mediator for AKH, but more research is needed to address this problem directly.

#### Insulin-Like Peptide 3

Mobilization of glycogen is a key process during diapause and starvation. Glycogen is primarily stored in the muscle, rather than the fat body, presumably to facilitate energy transport to tissues responsible for flight and evasion (Veenstra et al., 2008). Glycogen is broken down to glucose and trehalose in response to perceived starvation through AKH, but can also occur in the fat body via *ilp3* in an AKH-independent manner (Yamada et al., 2018). Perception of dietary sugars stimulates ILP3 secretion to activate fat body mTOR (Kim and Neufeld, 2015; Yamada et al., 2018). Work from Varghese et al. (2010), also implicates ILP3 in lipid homeostasis. *ilp3* overexpression is sufficient to rescue hyperlipidemia in *miR-14* mutant flies, though this function has not been further researched (Varghese et al., 2010). Interestingly, *ilp3* expression decreases with age, whereas most ILPs are known to increase (Tanabe et al., 2017). According to one study, *ilp3* is modestly increased in diapause (Kučerová et al., 2016). However, separate work demonstrates that mutants lacking *ilp2*, *ilp3*, and *ilp5* are more prone to diapause induction (Kubrak et al., 2014; Schiesari et al., 2016). Clearly, the function of ILP3 is dependent on competing signals in ways we do not yet understand. The age-associated changes in ILP3 will be of particular interest to the biology of aging, warranting further inspection into the opposing expression trajectories between old age and diapause.

#### Insulin-Like Peptide 8

ILP8 is relaxin-like and binds the leucine-rich repeat-containing G protein-coupled receptor 3 (Lgr3), as well as InR (Colombani et al., 2015; Garelli et al., 2015; Vallejo et al., 2015). Lgr3 is expressed throughout the organism, including the plasma membranes of the IPCs where it can be bound to modify insulin signaling. *ilp8* expression typically follows the metamorphic peaks of ecdysone, but is also induced through JNK signaling coordinated by Eiger, the TNFα ligand homolog in *Drosophila* (Sanchez et al., 2019). The latter scenario occurs in the imaginal disks through disturbance of tissue growth via genetic or mechanical injury to the organism during development (Colombani et al., 2012; Garelli et al., 2012; Ray and Lakhotia, 2017; Gontijo and Garelli, 2018). ILP8 delays development by inhibiting the production of PTTH, thereby modifying the release of ecdysone and allowing time to repair the damaged tissue before progressing to the next molt (Colombani et al., 2015; Ray and Lakhotia, 2017; Gontijo and Garelli, 2018). The role of ILP8 in diapause has not yet been explored, but as a stress-responsive peptide with the ability to delay development and upregulate repair processes, it is worthwhile to consider ILP8 in the diapause context. Accordingly, *ilp8* supports survival during starvation and regulates reproduction in young adults (Ables et al., 2016). Loss of *ilp8* reduces fecundity, though this appears to be caused by a defect in oviposition, rather than ovarian development (Liao and Nässel, 2020). In contrast, a separate study found that activation of *Lgr3*-expressing neurons in the abdominal ganglion of female flies decreases fecundity and discourages mating behavior (Meissner et al., 2016). The discrepancy is likely due to the difference in signaling locale, as well as the unknown molecular events that ILP8 causes by binding to InR. Taken together, ILP8 controls characteristics of diapause at various developmental stages. ILP8 may also contribute to longevity during diapause through its known roles increasing stress resistance. More research is needed to determine the place of ILP8 in the regulation of lifespan.

#### Insulin-Like Peptide 5

Although little is known about the function of ILP5, the majority of published work suggests it responds in a manner similar to ILP2 (Géminard et al., 2009; Wigby et al., 2011; Bai et al., 2012; Schiesari et al., 2016; Toprak, 2020). However, ILP5 is unique in certain respects. *ilp5* is expressed in the IPCs, as well as the renal tubes and ovarian follicles, whereas *ilp2* is excluded from these tissues (Ikeya et al., 2002; Okamoto et al., 2009; Söderberg et al., 2011). Intriguingly, *ilp5* is the only *Drosophila* ILP that is strongly reduced under dietary restriction (Min et al., 2008; Bai et al., 2012). This effect appears in larvae, IPCs, and principal cells of the renal tubes (Min et al., 2008; Géminard et al., 2009; Bai et al., 2012; Okamoto and Nishimura, 2015). In the IPCs, ILP5 becomes sequestered during dietary restriction while its mRNA transcript decreases (Géminard et al., 2009). mRNA levels of *ilp5* have not yet been tested in the renal tubes, but ILP5 itself is shown to decrease in this tissue (Söderberg et al., 2011). Loss of *ilp5* is correlated with, but does not cause, the lifespan extension phenotype observed with dietary restriction (Min et al., 2008). Rather, recent work shows that *ilp5* supports proper growth during poor nutrient conditions, inviting the possibility of a role for ILP5 in diapause (Okamoto and Nishimura, 2015). ILP5 mediates growth in response to the presence of amino acids in the fat body (Min et al., 2008; Broughton et al., 2010; Okamoto and Nishimura, 2015; Post and Tatar, 2016). It follows that *ilp5* expression is negatively regulated by FoxO and ILP6, two factors expressed during diapause and other non-feeding stages. Repression of *ilp5* may constitute part of a metabolic signaling pathway responsible for diapause induction. Supporting this, *ilp5*-null mutants have delayed time to pupariation (Okamoto and Nishimura, 2015). Loss of *ilp5* is also associated with reduced mating physiology and behavior, as well as increased survival during desiccation and oxidative stress (Söderberg et al., 2011; Wigby et al., 2011; Schiesari et al., 2016). *ilp5/ilp2* double mutants exhibit enhanced diapause inducibility, though this effect was not tested in single mutants, demonstrating the need for more direct evidence about the function of ILP5 in diapause (Schiesari et al., 2016).

#### Insulin-Like Peptides 7 and 4

Least is known about ILP7 and especially ILP4. Recent work shows that, in addition to ILP8, ILP7 is able to bind Lgr3 (Meissner et al., 2016; Prince et al., 2021). ILP7 acts upstream of IPC-derived ILPs to maintain IIS while the availability of dietary amino acids is low. This pathway is particularly relevant during diapause conditions which do not support the proliferation of protein-rich food sources (yeast on rotting fruit). Indeed, *Drosophila* show preference for plant-based food sources under conditions of cold temperatures (Brankatschk et al., 2014, 2018). Therefore, it may be the case that ILP7 plays a similar, yet distinct role to ILP5. ILP7 may regulate lifespan in diapause through upstream modulation of IPC-derived ILPs such as the pro-aging ILP2 or the pro-longevity ILP1.

### Concluding Statement on Insulin

Though diapause preparations help shield the organism from environmental stress, ILP signaling must remain dynamic throughout the phases of diapause to accommodate challenges both expected and unexpected (Denlinger, 2002; Sim and Denlinger, 2013; Chatterjee et al., 2014; Kubrak et al., 2014). This statement contrasts previous assertions that IIS is generally suppressed in diapause. However, as research on diapause has developed, more studies have examined IIS from a greater diversity of angles, creating a more complicated and realistic perspective of diapause regulation. The physiological state at the time of diapause induction is not the same as the state throughout, nor upon termination. Recent work suggests that diapause is best construed as an alternative phase of life during which energy is not constantly conserved, but metabolized in a more judicious way.

## Adipokinetic Hormone

Adipokinetic hormone, the functional insect homolog to glucagon, is responsible for the mobilization of energy stores from the fat body (Isabel et al., 2005; Mochanová et al., 2018). During early phases of *Drosophila* diapause, *akh* mRNA increases as mRNA levels of *ilp2*, *ilp3*, and *ilp5* decrease (Kubrak et al., 2014; Sinclair and Marshall, 2018; Post et al., 2019). The corresponding metabolic effects of these changes be observed in IPC-ablated flies, suggesting that the transcriptional profile induced via IPC ablation may share certain features with that of diapause (Ikeya et al., 2002; Reyes-DelaTorre et al., 2012). Indeed, genetic upregulation of AKH has been observed to cause similar phenotypes as in diapause, including longevity and starvation resistance (Waterson et al., 2014; Kučerová et al., 2016; Hughson et al., 2021). Though the diapause-associated increase in AKH might be seen as counterintuitive, given the typical diapause preparatory accumulation of energy stores, AKH is likely upregulated in early diapause to support the production of trehalose (Isabel et al., 2005; Hahn and Denlinger, 2011; Li et al., 2015). Trehalose is glucose disaccharide that functions as a cryoprotectant and energy source in diapause (Elbein et al., 2003; Ohtake and Wang, 2011; Teets and Denlinger, 2013). Additionally, trehalose is reported to induce autophagy and longevity, and confer resistance to infection and desiccation in non-insect models (Kyryakov et al., 2012; Tapia et al., 2015; Belzile et al., 2016; Rusmini et al., 2018; Seo et al., 2018; Yu et al., 2021). In insects, however, there is currently a lack of evidence to definitively determine whether trehalose confers the longevity associated with *akh* expression (Broughton et al., 2008). This discrepancy may be explained by the fact that the synthesis of trehalose is also supported by FoxO; instead of AKH, FoxO may upregulate trehalose production in parallel to other FoxO-dependent, beneficial effects observed in non-insect models (Hibshman et al., 2017). Alternatively, FoxO may promote trehalose production (or longevity) through the upregulation of AKH, which could confer longevity through means other than trehalose (Bednářová et al., 2015, 2018). One proposed mechanism involves Target of Brain Insulin (Tobi) (Buch et al., 2008). Tobi is an AKH-responsive alpha-glucosidase responsible for hydrolyzation of advanced glycation end products (AGEs), which are associated with diseases of old age in various model organisms (Oudes et al., 1998; Semba et al., 2010; Chaudhuri et al., 2018). In a transcriptomic study examining the mechanism of longevity in E(z) mutants, *tobi* was the most highly differentially expressed gene. Despite this, overexpression of *tobi* was found to diminish lifespan in flies (Buch et al., 2008; Moskalev et al., 2019).

The molecular mechanism underlying the pro-longevity effects of AKH in insects remains mysterious. Bednářová et al. (2015) showed this is achieved at least in part through activation of FoxO, indicating that diapause-like phenotypes induced by AKH are coordinated through feedback with IIS. During development, AKH is secreted from a subset of cells in the *corpora cardiaca* during periods of perceived nutritional deficiency to maintain metabolic homeostasis and resist starvation (Sinclair and Marshall, 2018). Recent work from Hughson et al. (2021), found that AKH during development helps regulate the timing of progression through molts by integrating environmental cues about nutrient availability. Authors observed that AKH signaling indeed participates in the endocrine regulation of development by signaling through projections of the *corpora cardiaca* that innervate the PG, suggesting additional interplay in developmental progress or diapause initiation through ecdysone. Supporting this notion, larvae without the AKH-responsive locus *dgc2* have delayed timing to pupariation when exposed to low nutrient conditions (Hughson et al., 2021).

## Juvenile Hormone

Though it has long been established that changes in the titers of juvenile hormones (JH) are associated with reproductive diapause, these studies are largely based on anatomical observations following injection or topical application (Denlinger et al., 2012). In most insect species, the induction of diapause coincides with a marked decrease in circulating JH (Agui et al., 1991; Hiroyoshi et al., 2017; Ma et al., 2021). In fewer species, however, diapause occurs with an increase in JH (Yin and Chippendale, 1973; Denlinger et al., 2012; Bebas et al., 2018). Research into the molecular regulation of JH in diapause is still in its infancy. JH is most well-studied during development as a metamorphic antagonist and activator of IIS (Minakuchi et al., 2008; Konopova et al., 2011; Kayukawa et al., 2016). The synthesis of JH takes place in the corpora allata (CA), a component of the ring gland that is retained in adulthood, under a complex and poorly understood regulatory network controlled by 20HE, as well as circadian rhythm-associated clock genes, allatotropin, allatostatin, and nutritional signals conveyed via IIS/TOR (Ikeno et al., 2010; Shin et al., 2012; Abdel-latief and Hoffmann, 2014; Kang et al., 2014; Ojima et al., 2018; Guo et al., 2021; Yang et al., 2021). From the CA, JH enters the hemolymph and travels to target tissues via juvenile hormone-binding protein, where it then utilizes the receptors Methoprene-tolerant (Met) and Germ cell-expressed (Gce), leading to the transcription and activation of anti-metamorphic targets such as Krüppel-homolog 1 and Hairy (Shin et al., 2012; Jindra et al., 2013; Gujar and Palli, 2016; Kayukawa et al., 2016; Zhang et al., 2019; Yang et al., 2021). Met is also capable of limiting metamorphic transcription further upstream through direct interaction with EcR and USP (Bitra and Palli, 2009; Zhao et al., 2014).

Upregulation of JH biosynthetic genes occurs in between molts and is responsible for the stimulation of growth to achieve critical mass (Truman and Riddiford, 2007; Borras-Castells et al., 2017). Conversely, JH is degraded rapidly at the onset of metamorphosis by a series of enzymes that convert active JH to JH acid diol and JH diol phosphate (Li et al., 2005; Lyu et al., 2019). Degradation of JH is also maintained during most instances of insect diapause, which has varied effects depending on the organism’s developmental stage (Denlinger et al., 2012; Fu et al., 2016; Tian et al., 2021). Specifically, the effects of low JH include the enhancement of innate immunity genes, desensitization to 20HE through reduction of a JH-inducible ecdysone competence factor, and the suppression of reproductive development and organismal growth via downregulation of IIS (Zhu et al., 2003, 2010; Flatt et al., 2008; Sheng et al., 2011; Mirth et al., 2014; Kayukawa et al., 2016; Borras-Castells et al., 2017). The JH-IIS axis has not yet been thoroughly explored, in spite of its relevance for both insect diapause and aging biology. In *Drosophila*, loss of JH signaling via CA ablation causes diapause phenotypes such as slow growth and longevity in a FoxO-dependent manner (Tatar et al., 2001; Mirth et al., 2014). Reciprocally, perturbations in IIS contribute to loss of JH (Tu et al., 2005). In *Aedes aegypti*, transcript levels of amino acid and sugar transporters are lower in diapause, likely contributing to reduced IIS through the low JH characteristic of diapause (Zhu et al., 2010). Evidence from tsetse flies also suggests an intersection of environmental stress, low JH and insulin, though more research is needed to understand how conserved the relationship between JH and IIS is across taxa (Baumann et al., 2013).

By contrast, the role of JH in reproduction is better understood. JH controls ovarian maturation in conjunction with ecdysone through the production of the oocyte yolk protein vitellogenin (Chinzei et al., 1982; Wyatt and Davey, 1996; Sheng et al., 2011; Yamamoto et al., 2013). This function was shown to be mediated by IIS in *Tribolium castaneum* (Sheng et al., 2011; Xu et al., 2013). It follows that the loss of reproduction in adult diapause is JH-dependent, although studies in *Colaphellus bowringi* and *Melinda pusilla* suggest JH alone is insufficient to control the non-reproductive adult diapause phenotype (Agui et al., 1991; Guo et al., 2019). Knockdown of diapause-associated JH degradation genes did not completely rescue ovarian development in diapause-destined *bowringi* females (Guo et al., 2019). Similarly, supplementation with JH analog restored ovarian development in diapausing *pusilla* females in a manner dependent on diapause phase (Agui et al., 1991). JH appears to be primarily regulated at the level of synthesis and degradation, though these results may still be explained through JH if downstream JH signaling components are lower in diapause (Gilbert et al., 2000; Tian et al., 2021). Of note is the observation that diapause traits other than reproduction were ameliorated upon knockdown of JH degradation genes, highlighting the expansive and complicated roles of JH in diapause. Complementing this idea, it has been shown that JH is also insufficient to control the timing of metamorphosis in early (larval) instars (Tan et al., 2005; Smykal et al., 2014). This may be related to the observation that diapause incidence in pre-adult molts tends to depend more on ecdysone than JH, assuming some mechanistic similarities between metamorphosis and diapause, although this relationship is not well conserved (Batz et al., 2019). In general, while the main outcomes of each insect endocrine molecule are similar across taxa, little is conserved in exactly how these hormones function together (Flatt et al., 2005; Mirth et al., 2014).

Among other diapause phenotypes, loss of JH increases lifespan in a multitude of species (Herman and Tatar, 2001; Yamamoto et al., 2013). Thus far little effort has been dedicated to understanding the molecular basis for longevity associated with low JH. It was previously accepted that longevity in diapause (and in broader contexts) resulted from a tradeoff with reproduction; the organism devotes energetic resources toward either maintenance of the gonads or the somatic tissues (Flatt and Kawecki, 2007). However, it has since been demonstrated that this “tradeoff” is coincidental, as lifespan can be uncoupled from reproductive capacity in plentiful ways (Corona et al., 2007; Yamamoto et al., 2013; Flatt and Partridge, 2018; Maklakov and Chapman, 2019; Moskalev et al., 2019). Given that diapause typically begins through downregulation of JH synthesis, it is reasonable to consider that loss of JH extends lifespan by inducing biological programs typically reserved for diapause. Yet, while genetic or surgical manipulations interrupt JH synthesis throughout the lifespan of the organism, levels of JH fluctuate during diapause (Denlinger et al., 1984; Jiang et al., 2011). Natural periods of JH elevation in diapause do not result in diapause termination and the organism remains extraordinarily long-lived, often far beyond the lifespan of insects unable to synthesize JH. Therefore, the JH-dependent mechanisms of longevity may borrow from, but do not replicate, mechanisms of longevity in diapause. Modification to IIS might represent one mode by which JH-null mutants exhibit extended lifespan in a way that borrows from programs of diapause (Cambron et al., 2021). As mentioned, the precise mechanisms linking JH signaling to IIS are poorly defined. Other modes include modifications to lipid metabolism, immunity, protein stability, mTOR, Jun-N-terminal kinase (JNK), or cell cycle/developmental programs dependent on Janus kinase/signal transducer and activator of transcription (JAK/STAT), all of which have been significantly noted in transcriptomic analyses of diapausing insects (Poelchau et al., 2013; Kubrak et al., 2014; Yocum et al., 2015; Kankare et al., 2016; Santos et al., 2018; Cambron et al., 2021). Unfortunately, these pathways have yet to be adequately explored at the intersection of diapause and lifespan. Employment of omics tools over the past several years has generated a wealth of information that will continue to provide avenues for future research into the molecular regulation of lifespan in diapause.

## Prothoracicotropic Hormone

Literature is scarce on the subject of prothoracicotropic hormone (PTTH) in diapause, but it is nonetheless important to discuss in this review for its intersection with more well-studied modes of diapause induction and exit. The traditional regulatory interaction between ecdysone and JH is mediated through PTTH and its receptor, Torso (Rewitz et al., 2009). As an activator of ecdysone biosynthesis, PTTH-producing neurons innervate the PG. Juvenile hormone binds and inhibits PTTH-producing neurons in between molts to limit the production of ecdysone. When JH is degraded at critical weight, PTTH-producing neurons become disinhibited, causing release of PTTH (Mirth et al., 2014). Subsequent binding to Torso located on the PG activates Ras/Raf/ERK signaling downstream, which increases intracellular cAMP to promote ecdysone biosynthesis.

However, recent work has prompted reanalysis of the canonical ecdysone synthesis pathway (Cruz et al., 2020; Pan and O’Connor, 2021). In both *Drosophila* and *Bombyx mori*, null mutants of PTTH or Torso will delay metamorphic transition from larvae to pupae, but will not arrest development indefinitely, implying the dispensability of PTTH for ecdysone biosynthesis in at least some insects (Uchibori-Asano et al., 2017). Similarly, about 50% of *Drosophila* with ablated PTTH-producing neurons will still undergo metamorphosis, albeit with significant delay. These findings strongly suggest another mode apart from PTTH is responsible for managing ecdysone at developmental transitions.

Cruz et al. (2020) have demonstrated that Egf signaling fills the dispensability gap of PTTH by activating Ras/Raf/ERK in parallel. Unlike *Ptth*-null mutants, *Egfr*-null mutants arrest development and do not resume. Importantly, this was shown to be dependent on ecdysone vesicle trafficking and biosynthesis. These results suggest Egf signaling may be the primary mode of regulation of ecdysone biosynthesis at developmental transitions. Additionally, Pan and O’Connor (2021) have identified several more candidate signaling pathways apart from PTTH to fine-tune metamorphic timing via ecdysone biosynthesis. Loss of anaplastic lymphoma kinase (Alk) and PDGF and VEGF receptor-related (Pvr) in the PG causes developmental delay in *Drosophila.* Ligands for these receptors originate from the PTTH-producing neurons, implying Alk, Pvr, and Torso have redundant functions in regulating ecdysone (Pan and O’Connor, 2021).

Under this model, PTTH is best construed as a means by which insects fine-tune the production of ecdysone in response to situational cues. This revision has clear implications for diapause, as one situation wherein environmental changes prompt adjustments to ecdysone signaling that occur outside the typical developmental program. This position is made stronger through the examination of previous work linking PTTH to growth regulators ILP8 and the glial amino acid transporter Sobremesa (Sbm) (Colombani et al., 2012; Galagovsky et al., 2018; Delanoue and Romero, 2020). Both ILP8 and Sbm suppress PTTH in response to various stressors to prevent metamorphosis under unfavorable conditions. ILP8 may also suppress PTTH during diapause, given the metabolic similarities between metamorphosis and diapause, as well as the fact that circulating PTTH is often low during diapause (Denlinger et al., 2012). Alternatively, in species that exhibit high ecdysone during diapause initiation, PTTH may serve to interrupt JH-dependent growth in between molts when Egf signaling is low.

To date, there has been no obvious role for PTTH in diapause, perhaps due to the inconsistent role of ecdysone across species. Recent advances have begun to paint a more complex and accurate picture of ecdysone regulation that will change the portrayal of PTTH in future literature.

## Future Directions and Conclusion

Diapause has evolved to incorporate information from every feature of seasonal change that could hinder the organism’s path to reproductive success. Considering this, the complex nature of the diapause endocrine program is to be expected. Peptide hormones from distant tissues must communicate with signaling programs elsewhere to coordinate responses in behavior, metabolism, growth, cuticle structure, dietary preferences, and more, in ways that generally run counter to the normal trajectory of development. Diapause cannot be achieved without careful timing and balance of hormonal transitions. This is best demonstrated through the well-documented developmental lethality of topical JH, or the many partial diapause phenotypes achievable through piecewise interventions in endocrine signaling (Spielman and Williams, 1966; Shaaya and Pisarev, 1986). Evolution has arrived at this balance on many occasions, given the different—and sometimes opposing—ways that different species initiate diapause. Indeed, the extension of diapause into vertebrate systems such as bats, rodents, fish, birds, bears and marsupials gives hope that later work will compare mechanisms of diapause across phyla (Ptak et al., 2012; Deng et al., 2018; Rasweiler and Badwaik, 2020).

Partial diapause is readily inducible through manipulations in IIS, diet, and other aspects of physiology. However, even interventions of major upstream regulators (InR deletion, IPC ablation, CA removal, CC stimulation), do not recapitulate diapause entirely. Diapause endocrinology is a study of interactions. It is not yet fully understood how most hormones interact throughout the phases of diapause, nor through which pathways, to arrive at homeostasis. Valuable studies have begun to connect these pieces, though more translational work is needed to construct a cohesive network of endocrine in diapause. In a similar sense, lifespan is a multi-faceted trait whose length depends on species-specific adaptations, in addition to environmental ones (Rasweiler and Badwaik, 2020). Much has been accomplished in recent years to characterize the molecular events responsible for these adaptations. Still, even more could be achieved through their examination in light of evolution. The aim of this review is to poise diapause endocrinology in a way that provides context to the question of how a species arrives at its normal lifespan. Diapause is a natural case of lifespan plasticity; the mechanisms that determine a species’ lifespan are dramatically altered in a very conserved manner, making them able to be probed scientifically. Without this context, discoveries about the determination of lifespan are rarer and more difficult to understand, as they lack the evolutionary perspective needed to describe the extension in a systemic way.

Canonical regulators of aging are implicated in diapause. Insofar as both aging and diapause involve changes to most aspects of physiology, significant overlap is expected. However, beyond the shared categories of physiology, the specific ways in which physiology is altered in diapause reflects physiological changes in laboratory extensions of lifespan. For example, somatic reduction of mTOR activity is known to extend lifespan outside the immediate context of diapause, and is also observed in diapause (Kapahi et al., 2004; Liu et al., 2017; Cohen, 2018). Other pathways beyond the scope of this review are regulated similarly, such as the retention of genomic stability or the induction of longevity-associated microRNAs (Saravanakumar et al., 2010; Liu et al., 2012; Vijg and Suh, 2013; Batz et al., 2017; Gendron and Pletcher, 2017; Reynolds et al., 2017). These similarities suggest that evolutionarily conserved aspects of diapause are the natural context for why laboratory manipulations extend lifespan and increase stress resistance. However, laboratory manipulations of lifespan do not replicate the extreme longevity of diapause. As mentioned, this may be attributed to the fact that laboratory manipulations of lifespan induce partial diapause, but fail to capture the intricate balance of hormones required for full diapause.

Other insect peptide hormones and neuropeptides that were not included in this review are likely regulated in diapause-specific manners (Nässel and Zandawala, 2020). However, less is known about the specific functions of these peptides in diapause. The purpose of this review is to speculate about roles in longevity for diapause-associated hormones; therefore, it would be inappropriate to do so using a peptide whose role in diapause is already speculative. The exception to this statement is the diapause hormone of *B. mori* (Hasegawa, 1964; Jiang et al., 2019). Secretion of diapause hormone is promoted in oocytes destined for diapause by environmental perception of the mother pre-deposition. Significant work has dissected the regulation of *B. mori* diapause in relation to numerous aspects of physiology, including other endocrine molecules, but thus far diapause hormone appears to be exclusive to moths (Ichikawa et al., 1997; Cui et al., 2021; Tsuchiya et al., 2021). While diapause hormone likely impacts lifespan, research in the biology of aging most clearly benefits from examination of mechanisms in diapause that are well-conserved. For this reason, diapause hormone is not examined here in the context of lifespan.

Diapause represents a fantastic model system to understand the complex intersection of environmental stimuli, endocrine regulation, development and longevity. From current literature, it can be inferred that a complete understanding of diapause requires integration of knowledge from domains in biology that are often studied separately. Advances in omics technology provide a highly informative and efficient means to accomplish this. An increasing number of omics experiments under various conditions relevant to, or directly observing, insect diapause are mapping the contributions of individual hormones to diapause traits. Comparing these studies with data from single interventions in longevity could help identify mechanisms of lifespan plasticity in diapause, while supplying evolutionary context for how lifespan is determined in non-diapausing species. However, metanalytical work such as this is rarely performed, and even within the same species, there is little consistency in experimental design and choice of analysis. These are obstacles that must be controlled for. Comparative omics could discern conserved mechanisms from non-conserved ones that define the diapause endocrine program across species. Future work will reveal much about how lifespan evolved as a plastic component in the larger system of diapause.

## Author Contributions

The author confirms being the sole contributor of this work and has approved it for publication.

## Conflict of Interest

The author declares that the research was conducted in the absence of any commercial or financial relationships that could be construed as a potential conflict of interest.

## Publisher’s Note

All claims expressed in this article are solely those of the authors and do not necessarily represent those of their affiliated organizations, or those of the publisher, the editors and the reviewers. Any product that may be evaluated in this article, or claim that may be made by its manufacturer, is not guaranteed or endorsed by the publisher.

## Abbreviations

JH: juvenile hormone
IIS: insulin/insulin-like signaling
AKH: adipokinetic hormone
PTTH: prothoracicotropic hormone
ILP: insulin-like peptide
20HE: 20-hydroxyecdysone
PG: prothoracic gland
CA: corpora allata
CC: corpora cardiaca
IPCs: insulin producing cells.
